# Complete Radiologic Response of Metastatic Pancreatic Ductal Adenocarcinoma to Microwave Ablation Combined with Second-Line Palliative Chemotherapy

**DOI:** 10.1155/2020/4138215

**Published:** 2020-01-31

**Authors:** Hakon Blomstrand, Karin Adolfsson, Per Sandström, Bergthor Björnsson

**Affiliations:** ^1^Department of Clinical Pathology and Department of Clinical and Experimental Medicine, Linköping University, 58183 Linköping, Sweden; ^2^Department of Oncology, Ryhov County Hospital, 55305 Jönköping, Sweden; ^3^Department of Surgery and Department of Clinical and Experimental Medicine, Linköping University, 58183 Linköping, Sweden

## Abstract

Pancreatic ductal adenocarcinoma (PDAC) has a bleak prognosis, especially for the majority of patients diagnosed with metastatic disease. The primary option for palliative treatment is chemotherapy, and responses beyond first-line treatment are rare and typically short. Here, we report a case of a 63-year-old woman with PDAC in the head of the pancreas who was initially successfully treated by pancreaticoduodenectomy followed by adjuvant chemotherapy with gemcitabine. However, disease recurrence with liver and para-aortic lymph node metastases was detected only two months after the completion of adjuvant chemotherapy. First-line palliative chemotherapy with gemcitabine-nab/paclitaxel was commenced. The results were discouraging, with disease progression (liver and lung metastases) detected at the first evaluation; the progression-free survival was just two months (64 days). Surprisingly, the response to second-line palliative chemotherapy with 5-fluorouracil-oxaliplatin was excellent; in combination with the ablation of a liver metastasis, this treatment regimen resulted in a complete radiological response and an 11-month treatment-free interval with a sustained good performance status.

## 1. Background

Pancreatic ductal adenocarcinoma (PDAC) is the fourth leading cause of cancer-related death in Sweden and the USA [1, 2]. Initial symptoms are often nonspecific, and most patients are diagnosed with locally advanced pancreatic cancer (LAPC) and/or metastatic disease.

PDAC without distant metastasis is divided into resectable PDAC, borderline resectable pancreatic cancer (BRPC), and LAPC, largely dependent on the extent of vessel involvement [3]. For resectable PDAC without vessel involvement, the standard treatment is initial surgery with pancreaticoduodenectomy or distal resection, depending on tumour location, followed by single-agent adjuvant chemotherapy with 5-fluorouracil (5FU) or gemcitabine [4]. This subgroup of PDAC patients has the best outcome, but the frequency of recurrence is high, and long-term survival at five years is only 15–20% [5]. Adjuvant combination chemotherapy with gemcitabine-5FU appears to improve outcomes [6], and neoadjuvant chemotherapy is being evaluated in several ongoing trials [7]. For the BRPC subgroup with tumour infiltration of local major vessels (mainly the celiac trunk, superior mesenteric artery, and hepatic artery), the risk of microscopically positive resection margins (R1 resection) following surgery is substantial; therefore, combination chemotherapy and multimodal treatments (e.g., chemotherapy with sequential or concomitant radiotherapy) have been tested in numerous trials, but the results on the most effective strategy are contradictory (reviewed in [8]). The LAPC subgroup with extensive vessel engagement has a high risk of macroscopically positive resection margins (R2 resection), and surgery should not be considered unless conversion to BRPC is achieved during palliative treatment [9].

In nonresponding LAPC or primary metastatic disease, resection of the primary tumour is not possible or not indicated, respectively, and the treatment option is palliative care. According to current guidelines, the only treatment modalities for patients with this disease are chemotherapy and radiation therapy for severe and refractory pain [10, 11]. The prognosis of patients with metastatic pancreatic cancer is poor, with an expected five-year survival rate of 8% in Sweden [12]. Histological subtypes other than ductal adenocarcinoma are rare, and signet ring cell carcinoma appears to have a similar prognosis [13], while the colloid variant (mucinous noncystic adenocarcinoma) has a markedly better prognosis [14].

## 2. Case Presentation

**Figure 1:**
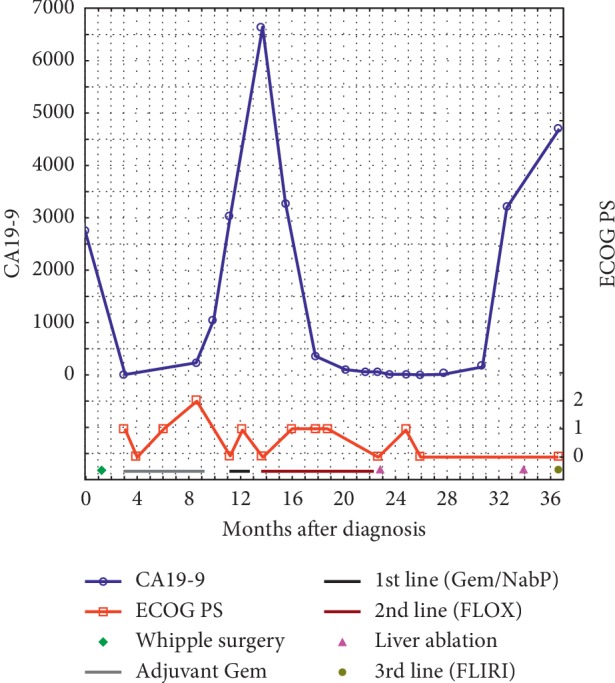
Variation over time in CA19-9 levels and the ECOG PS in relation to treatment. Abbreviations: ECOG, Eastern Cooperative Oncology Group; PS, performance status; Gem, gemcitabine; NabP, nab-paclitaxel; FLOX, bolus 5-FU plus oxaliplatin; FLIRI, bolus 5-FU plus irinotecan.

**Figure 2:**
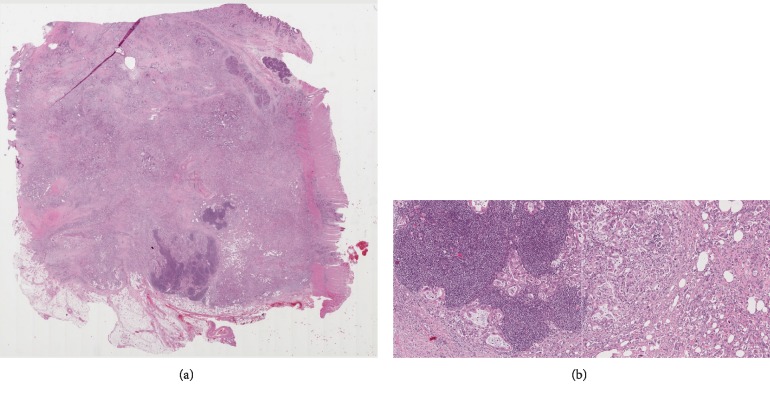
Histologic haematoxylin- and eosin-stained paraffin section of the patient's primary tumour (a) and detailed image of lymphatic invasion and tumour morphology (b).

A 63-year-old Swedish woman consulted her local health centre due to nausea, bright coloured stools, and 4 kg weight loss. Laboratory tests revealed elevated aminotransferases and bilirubinaemia, and the patient was referred to the local surgical department. Abdominal ultrasound and computed tomography (CT) of the abdomen and thoracic cavity revealed a tumour in the pancreatic head measuring 25 mm, a borderline enlarged lymph node in the liver hilum, and dilation of the pancreatic and common bile ducts but no evidence of distant metastases. CA19-9 was elevated to 2741 kU/L (<35 kU/L) (Figure 1). Internal bile duct drainage via endoscopic retrograde cholangiopancreatography (ERCP) failed; thus, the patient underwent percutaneous transhepatic cholangiography (PTC) drainage and was treated twice for cholangitis with intravenous antibiotics before undergoing surgery at the regional university hospital. The surgical procedure was a standard pancreaticoduodenectomy with an uneventful recovery. The patient was discharged to the local hospital on the fourth postoperative day (POD) and to the home on POD 15. The histopathological examination confirmed moderately to poorly differentiated PDAC. Although minor sections of the tumour had mucinous and signet ring cell-like histology, the diagnosis was not changed. Perineural and lymphovascular invasion was evident. Carcinoma involvement was seen in two pancreaticoduodenal lymph nodes out of a total of 20 examined lymph nodes. The tumour grade was pT3N1MX (Figure 2). Postoperative CA19-9 was 6 kU/L. Six months of adjuvant treatment with gemcitabine (intravenous infusions on days 1, 8, and 15 in a four-week cycle at 1000 mg/m^2^) was initiated. The patient completed the entire treatment regimen, except the dose on cycle 4 day 15 due to *E. coli* septicaemia, which was successfully treated with antibiotics. At the evaluation at the end of adjuvant treatment, 7.5 months after surgery, there were no signs of radiological recurrence on CT; however, CA19-9 was increased to 229 kU/L.

**Figure 3:**
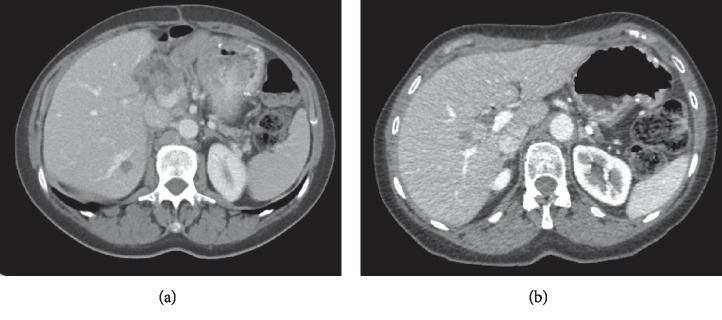
Liver metastases: the lesion in segment 6 was treated with microwave ablation (a), and the lesion in segment 5 was treated with RFA (b).

**Figure 4:**
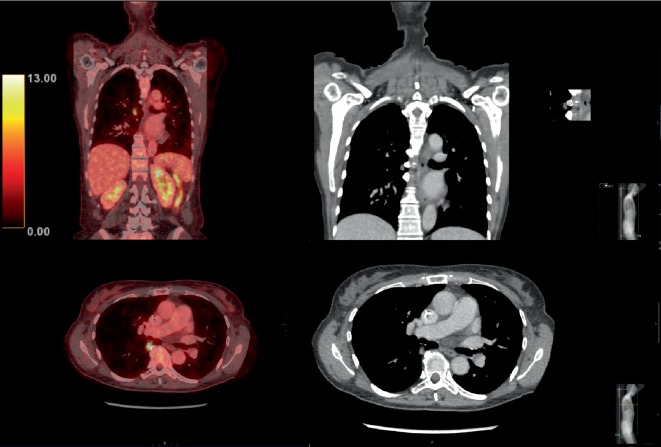
PET-CT evaluation of the response to first-line palliative chemotherapy showed disease progression with lung metastasis. PET-CT (left column) and CT (middle column) images. Top right inset: higher magnification image and measurement of the lesion. The SUVmax of the lesion was 9.7.

**Figure 5:**
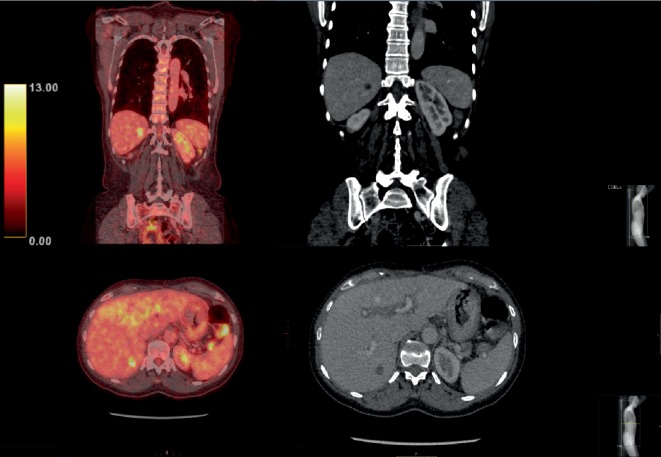
No change in the liver metastasis in segment 6 was observed upon evaluation of the response to first-line palliative chemotherapy. PET-CT (left column) and CT (right column) images. The SUVmax was 5.5 for the lesion and 3.1 for the surrounding liver.

**Figure 6:**
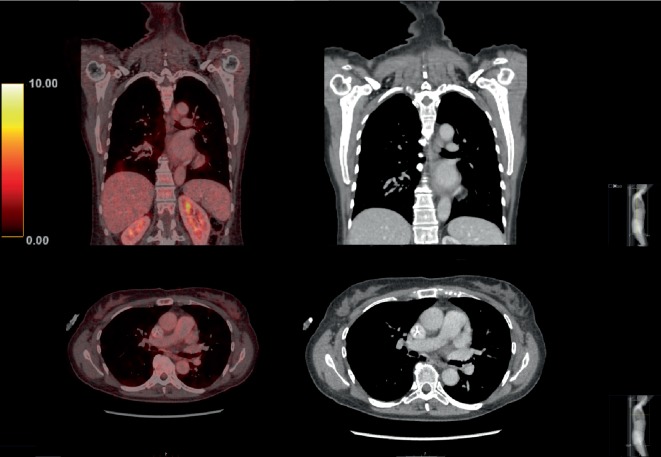
PET-CT images showing a complete radiological response of the lung metastasis. PET-CT (left column) and CT (right column) images.

After an additional two months of observation, CA19-9 increased to 3026 kU/L, and CT revealed one liver metastasis in segment 6 (Figure 3) and multiple enlarged para-aortic lymph nodes. The patient was treated with two full cycles of gemcitabine/nab-paclitaxel as first-line palliative chemotherapy according to regional guidelines. The treatment was administered by intravenous infusions on days 1, 8, and 15 in a four-week cycle at 1000 mg/m^2^ gemcitabine and 125 mg/m^2^ nab-paclitaxel. The response was evaluated with positron emission tomography (PET)-CT, which revealed no change in the liver metastasis in segment 6 or in lymph nodes without uptake; however, a new paravertebral tumour with FDG uptake was detected in the lower right lobe of the lungs (Figures 4 and 5). CA19-9 was 6628 kU/L. The diagnosis was progressive disease, and second-line palliative 5FU-based chemotherapy was commenced. The first cycle was given as FLV (500 mg/m^2^ 5FU bolus plus 60 mg/m^2^ leucovorin on days 1 and 2 in a two-week cycle) due to persistent chemotherapy-induced polyneuropathy (CIPN) from the first-line treatment. The CIPN decreased to CTCAE grade 1, allowing for the administration of FLOX (FLV plus the infusion of 85 mg/m^2^ oxaliplatin on day 1 of the two-week cycle) from the second cycle. Repeated CT scans showed a partial response, and CA19-9 decreased to 44 kU/L during 17 cycles over a 9-month treatment period. The oxaliplatin dose was reduced because of CIPN and discontinued from cycle 14 due to an adverse drug reaction; drug hypersensitivity could not be ruled out. PET-CT and contrast-enhanced ultrasound of the liver showed a complete response in the thoracic cavity (Figure 6) and one 13-mm liver metastasis in segment 6 as the only remaining radiological evidence of disease.

Due to the earlier adverse reaction to oxaliplatin, persistent CIPN, and chemotherapy-related fatigue, continued palliative chemotherapy was difficult and had no curative potential. In part based on the patient's request, alternative treatment strategies were considered, and local treatment with microwave ablation (MWA) was offered after discussion at a multidisciplinary conference. The patient underwent MWA of the liver lesion 22 months after surgery for the primary tumour, and CA19-9 levels were normalized after MWA. Due to the presence of a postablation liver abscess, continued chemotherapy was contraindicated, and an active monitoring strategy was chosen. The patient was evaluated every second month and showed a slow increase in CA19-9 to 151 kU/L by 30 months after surgery for the primary tumour but no radiological evidence of relapse. CA19-9 further increased to 3182 kU/L after an additional 2 months, and PET-CT showed one liver metastasis in segment 5 measuring 15 mm (Figure 3) without additional dissemination; thus, the patient, after discussion at a multidisciplinary conference, underwent successful radiofrequency ablation (RFA) 33 months after surgery for the primary tumour.

At the next evaluation visit 3 months later (i.e., 36 months after primary surgery), a relapse was found both biochemically, with a further increase in CA19-9 to 4692 kU/L, and radiologically, with PET-CT showing three suspicious 4 to 8 mm lung metastases without FDG uptake and two metastases in the right liver lobe measuring 25 and 30 mm. Third-line palliative chemotherapy with FLIRI (FLV plus the intravenous infusion of 180 mg/m^2^ irinotecan on day 1 in the two-week cycle) was started.

Except during the initial adverse event of a liver abscess following the MWA, which was successfully treated with antibiotics, the patient did not suffer from any side effects after local treatment. During the eleven-month relapse-free interval and until the start of third-line palliative chemotherapy with FLIRI, the patient reported a WHO performance status of 0. The patient experienced normal health and pursued physical activity and hobbies as before the cancer diagnosis.

## 3. Discussion

The frequently observed course of metastatic PDAC is rapid progression, but there is now a fairly good chance for a response to first-line palliative combination chemotherapy [15]; in the South East Region of Sweden, the median overall survival of this patient group is approximately 9 months [16]. Based on the results of the phase III MPACT trial [15], many guidelines advocate the use of gemcitabine-nab/paclitaxel as first-line palliative treatment in patients with metastatic PDAC and ECOG PS 0-1, as is the case in the South East Region of Sweden. This case illustrates that even in PDAC, some patients who are nonresponders to the recommended treatment do experience a response to alternative chemotherapy treatments, indicating the need for therapy-specific predictive factors to optimize oncologic treatment decision-making.

In treating patients with metastatic PDAC with second- or even third-line chemotherapy, there is a fine balance between patient frailty and drug toxicity. In Sweden, platinum-based combination chemotherapy (in this case, FLOX) is often offered to patients with a good performance status based on the survival benefit seen in previous small phase II studies and a phase III study [17]. These data have since been questioned as the results from a more recent phase III study were contradictory, with a shorter median OS in the platinum-based therapy (FOLFOX) group than in the FLV only group [18]. In addition, the objective response rate on second-line therapy is often less than 10% [19], indicating the importance of appropriate patient selection. As predictors of the outcome of individual chemotherapy regimens are lacking, clinical parameters and the ECOG or Karnofsky performance status are often used, and a nomogram to help choose the right patients for second-line treatment has been proposed [20].

With the abovementioned data in mind, this case illustrates an example of an exceptional response to second-line palliative chemotherapy. Although limited conclusions can be drawn from a single case, this patient had several prognostic factors associated with a poor outcome in the previously reported nomogram, such as liver metastasis, advanced age, and a very short duration of first-line chemotherapy. The factors that contributed to the therapeutic response in this patient are unknown, but the current report illustrates that second-line therapy can be effective and should be considered, even in motivated patients with multiple aggravating clinical factors.

In metastatic PDAC, clinical guidelines do not include surgery or local treatment modalities, which is in contrast to colorectal cancer, for which the surgical approach (with a curative intention) to liver and lung metastases is routine [21, 22]. For PDAC, the ablative modality of irreversible electroporation (IRE) has been evaluated as a first-line treatment for LAPC but has not shown a significant survival benefit [23]. IRE could theoretically be used for hilar liver metastases [24], but this was not an option in this case due to the location of the liver metastasis. Concerning the choice between RFA or MWA, data on hepatocellular carcinoma and colorectal liver metastases suggest similar efficacy, with a tendency towards better local control with MWA, especially in cases of large lesions [25, 26]. In the case presented herein, ablation of the liver metastasis was used in conjunction with chemotherapy and achieved an 11-month treatment-free interval without radiologically detectable disease and with a subjective and objective (WHO PS) good quality of life. The duration was far longer than expected, considering the unambiguous histopathological diagnosis of PDAC and the immediate progression on first-line palliative chemotherapy. This, together with the recurrence in another liver lobe and the absence of evidence for the curative potential of chemotherapy in metastatic PDAC, points towards an actual treatment effect of MWA. Though not possible in this case due to infectious complications of MWA, the effect could hypothetically have been prolonged by “adjuvant” chemotherapy, considering the previous response to second-line chemotherapy. However, as the overall intention was palliative, the value of the treatment-free period should not be underestimated.

The choice of a second liver ablation over chemotherapy was based on the treatment-free interval after the initial ablation, the good quality of life reported by the patient in the chemotherapy-free interval, and the radiological finding of a single new lesion in the liver.

## 4. Conclusion

This case illustrates that a treatment-free interval with a sustained good performance status can be achieved with second-line palliative chemotherapy in combination with the ablation of liver metastases in patients with pancreatic cancer and that local therapies should therefore be considered in select cases.

## References

[1] Siegel R. L., Miller K. D., Jemal A. (2017). Cancer statistics, 2017. *CA: A Cancer Journal for Clinicians*.

[2] Bergman O., Jaresand M. (2018). *Cancerfondsrapporten*.

[3] Katz M. H. G., Pisters P. W. T., Lee J. E., Fleming J. B. (2011). Borderline resectable pancreatic cancer: what have we learned and where do we go from here?. *Annals of Surgical Oncology*.

[4] Neoptolemos J. P., Stocken D. D., Bassi C. (2010). Adjuvant chemotherapy with fluorouracil plus folinic acid vs gemcitabine following pancreatic cancer resection. *JAMA*.

[5] Hishinuma S., Ogata Y., Tomikawa M., Ozawa I., Hirabayashi K., Igarashi S. (2006). Patterns of recurrence after curative resection of pancreatic cancer, based on autopsy findings. *Journal of Gastrointestinal Surgery*.

[6] Neoptolemos J. P., Palmer D., Ghaneh P. (2016). ESPAC-4: a multicenter, international, open-label randomized controlled phase III trial of adjuvant combination chemotherapy of gemcitabine (GEM) and capecitabine (CAP) versus monotherapy gemcitabine in patients with resected pancreatic ductal adenocarcinoma. *Journal of Clinical Oncology*.

[7] Silvestris N., Longo V., Cellini F. (2016). Neoadjuvant multimodal treatment of pancreatic ductal adenocarcinoma. *Critical Reviews in Oncology/Hematology*.

[8] Silvestris N., Brunetti O., Vasile E. (2017). Multimodal treatment of resectable pancreatic ductal adenocarcinoma. *Critical Reviews in Oncology/Hematology*.

[9] Tol J. A. M. G., Eshuis W. J., Besselink M. G. H., van Gulik T. M., Busch O. R. C., Gouma D. J. (2015). Non-radical resection versus bypass procedure for pancreatic cancer—a consecutive series and systematic review. *European Journal of Surgical Oncology (EJSO)*.

[10] Ducreux M., Cuhna A. S., Caramella C. (2015). Cancer of the pancreas: ESMO Clinical Practice Guidelines for diagnosis, treatment and follow-up. *Annals of Oncology*.

[11] Tempero M. A., Malafa M. P., Al-Hawary M. (2017). Pancreatic adenocarcinoma, version 2.2017, NCCN clinical practice guidelines in oncology. *Journal of the National Comprehensive Cancer Network*.

[12] Bergman O., Johansson E. (2018). *Cancer I Siffror 2018*.

[13] Patel M. (2018). The impact of epidemiological factors and treatment interventions on survival in patients with signet ring cell carcinoma of the pancreas. *American Journal of Clinical Oncology*.

[14] Poultsides G. A., Reddy S., Cameron J. L. (2010). Histopathologic basis for the favorable survival after resection of intraductal papillary mucinous neoplasm-associated invasive adenocarcinoma of the pancreas. *Annals of Surgery*.

[15] Von Hoff D. D., Ervin T, Arena F. P (2013). Increased survival in pancreatic cancer with nab-paclitaxel plus gemcitabine. *The New England Journal of Medicine*.

[16] Blomstrand H., Scheibling U., Bratthäll C., Green H., Elander N. O. (2019). Real world evidence on gemcitabine and nab-paclitaxel combination chemotherapy in advanced pancreatic cancer. *BMC Cancer*.

[17] Oettle H., Riess H., Stieler J. M. (2014). Second-line oxaliplatin, folinic acid, and fluorouracil versus folinic acid and fluorouracil alone for gemcitabine-refractory pancreatic cancer: outcomes from the CONKO-003 trial. *Journal of Clinical Oncology*.

[18] Gill S., Ko Y.-J., Cripps C. (2016). PANCREOX: a randomized phase III study of fluorouracil/leucovorin with or without oxaliplatin for second-line advanced pancreatic cancer in patients who have received gemcitabine-based chemotherapy. *Journal of Clinical Oncology*.

[19] Rahma O. E., Duffy A., Liewehr D. J., Steinberg S. M., Greten T. F. (2013). Second-line treatment in advanced pancreatic cancer: a comprehensive analysis of published clinical trials. *Annals of Oncology*.

[20] Vienot A., Beinse G., Louvet C. (2017). Overall survival prediction and usefulness of second-line chemotherapy in advanced pancreatic adenocarcinoma. *JNCI: Journal of the National Cancer Institute*.

[21] Lykoudis P. M., O’Reilly D., Nastos K., Fusai G. (2014). Systematic review of surgical management of synchronous colorectal liver metastases. *British Journal of Surgery*.

[22] Sun Z., Thacker J. M. (2015). Contemporary surgical options for metastatic colorectal cancer. *Current Oncology Reports*.

[23] Månsson C., Brahmstaedt R., Nygren P., Nilsson A., Urdzik J., Karlson B.-M. (2019). Percutaneous irreversible electroporation as first-line treatment of locally advanced pancreatic cancer. *Anticancer Research*.

[24] Scheffer H. J., Melenhorst M. C. A. M., Echenique A. M. (2015). Irreversible electroporation for colorectal liver metastases. *Techniques in Vascular and Interventional Radiology*.

[25] Facciorusso A., Di Maso M., Muscatiello N. (2016). Microwave ablation versus radiofrequency ablation for the treatment of hepatocellular carcinoma: a systematic review and meta-analysis. *International Journal of Hyperthermia*.

[26] Takahashi H., Kahramangil B., Kose E., Berber E. (2018). A comparison of microwave thermosphere versus radiofrequency thermal ablation in the treatment of colorectal liver metastases. *HPB*.

